# Investigating perspective taking and caregiver-proxy–child communication attitude agreement in early childhood stuttering

**DOI:** 10.1371/journal.pone.0339706

**Published:** 2026-02-12

**Authors:** Katie L. Winters, Courtney T. Byrd

**Affiliations:** 1 Department of Communication Sciences & Disorders, University of Cincinnati, Cincinnati, Ohio, United States of America; 2 Department of Speech, Language, Hearing Sciences, The University of Texas at Austin, Austin, Texas, United States of America; University of Montreal: Universite de Montreal, CANADA

## Abstract

**Purpose:**

Children begin to stutter and develop early attitudes about their communication during a critical period of speech, language, and social cognition development. This study examined social cognition via cognitive and affective perspective taking and explored whether perspective taking influences caregiver-proxy – child communication attitude agreement in 3–6-year-old children who stutter, while accounting for variation in speech and language abilities.

**Method:**

One hundred eleven children who stutter, ages 3–6, completed the *KiddyCAT,* protocols for cognitive and affective perspective taking, and standardized speech and language assessments. One primary caregiver completed an adapted version of the *KiddyCAT*, which involved estimating their perception of their child’s communication attitude. Analyses were conducted to calculate the caregiver’s item-by-item agreement with their child’s responses on the *KiddyCAT*, and multiple regression was used to assess predictors of caregiver-proxy – child *KiddyCAT* agreement.

**Results:**

Approximately 8% of children performed above chance on the cognitive perspective taking task, whereas 56% performed above chance on the affective task. Caregiver-proxy – child *KiddyCAT* agreement ranged from 0.56–0.85, with higher agreement on two items requiring the child’s perspective taking. Higher speech and language scores predicted greater *KiddyCAT* agreement for all caregiver-child dyads. Neither affective nor cognitive perspective taking predicted *KiddyCAT* agreement.

**Conclusions:**

Consistent with research with typically developing children, there is substantial variability in perspective taking in children age 3–6 who stutter, with evidence of emerging affective, but comparatively limited cognitive perspective taking skills. Caregiver-proxy – child agreement on the Kiddy-CAT, a measure that requires both affective and cognitive perspective taking, appears to be stronger for children with more advanced speech and language. Given that at least some young children and their caregivers appear to be aware of each other’s perspectives regarding the child’s communication, clinicians should facilitate discussion of stuttering and reactions to stuttering openly and neutrally with these caregivers and their children.

## Introduction

Near the typical onset of stuttering, children begin developing the ability to take another individual’s perspective and they also begin to form attitudes towards their communication [1–5]. Communication attitude is commonly defined as the learned predisposition to evaluate and respond to communicative interactions in either a positive or negative manner [6]. Given that many clinicians rely on caregiver reports to assess a child’s communication and the evidence to suggest that a child’s self-perception may be significantly influenced by caregivers’ opinions [7–9], more exploration is warranted specific to agreement between caregiver-proxy and child reports of communication attitudes (referred to throughout this study as “caregiver-proxy – child agreement”). Additionally, although studies have demonstrated that children with speech and language disorders tend to exhibit more negative communication attitudes [10] and that limited language skills may restrict perspective taking [11], no study has yet examined how perspective taking uniquely influences caregiver-proxy – child agreement on these attitudes in children who stutter while accounting for their speech and language skills. Accounting for these skills is critical as their speech and/or language abilities may distinctly influence attitudinal assessment scores, particularly on any aspect of an assessment that requires the child to adopt the perspective of others, further highlighting a significant gap in the literature. Thus, to advance understanding of the communication attitudes of children who stutter, there is a need for research that simultaneously considers perspective taking and speech–language abilities when examining caregiver-proxy – child communication attitude agreement.

### Childhood stuttering

Childhood stuttering most often begins between ages 2 and 6, at which time many children develop an awareness that they stutter and a positive, neutral, or negative communication attitude [1,2,4]. Numerous studies demonstrate 3–6-year-old children who stutter across cultural and linguistic backgrounds report more negative communication attitudes compared to same-age children who do not stutter on the KiddyCAT [4,12–17]. Additional studies have demonstrated that caregivers also report their young children who stutter are both aware of and negatively impacted by their stuttering [2,18,19]. Notably, these negative attitudes reported by caregivers and their children who stutter are independent from characteristics of their overt stuttered speech. Specifically, high stuttering frequency is not associated with more negative communication attitudes, and low stuttering frequency is not predictive of more positive communication attitudes [19,20]. Thus, consistent with general attitude development [6,21–23], a young child’s communication attitude is not anchored to the behavior of stuttering only, but, like general attitudes, also stems from their affective and cognitive experiences related to the child’s overall communication.

Stuttering onset and development of children’s initial communication attitudes accompany a critical developmental period for a child’s speech and language [24–28]. Prior research investigating the speech sound or language skills of children who stutter suggests that some children present with skills that meet or exceed typical age expectations, while others present with concomitant diagnoses such as a language disorder or speech sound disorder [3,28–31]. For example, a meta-analysis by Ntourou and colleagues [28] suggested some children who stutter present with subtle language differences (i.e., not language disorders), while Arndt and Healey [29] reported 44% of children who stutter who received clinical services from a speech-language pathologist also had a co-occurring phonological and/or language disorder. Similarly, clinical prevalence of co-occurring stuttering and speech sound disorders has been reported to be between 6.88% and 46% of children who receive intervention services for stuttering [31]. In contrast to stuttering frequency data and the communication attitudes of children who stutter, research suggests there is a correlation between speech sound disorder severity and communication attitude: children with more noticeable speech sound disorders – characterized by lower percent consonants correct – also report more negative communication attitudes [10].

To date, to our knowledge, there is no published research investigating communication attitudes of children who stutter wherein their speech and language abilities are accounted for in the analyses and related interpretations. Such exploration is critical given the data to demonstrate that children with speech sound as well as children with language deficits may have more negative communication attitudes, and the data to suggest that the ability to demonstrate perspective taking, a skill that is required on one of the most commonly used assessments of communication attitude, may be uniquely influenced by their language ability.

### Perspective taking

Perspective taking is one component of social cognition that refers to the ability to identify another person’s thoughts and feelings as distinct from one’s own. Prior to age 2, children begin to identify and respond to others’ emotions, even when those emotions are not expressed overtly through verbal or nonverbal communication [32]. By ages 3 and 4, children recognize external factors that might influence someone’s emotions, and they anticipate others’ feelings when something negative happens [33]. By preschool, children further develop their ability to make inferences about others’ thoughts (i.e., cognitive perspective taking) and emotions (i.e., affective perspective taking) [24,26].

Cognitive perspective taking is sometimes referred to as Theory of Mind [34–36]. Previous research has evaluated cognitive perspective taking via false belief understanding tasks using a variety of experimental paradigms, such as location change tasks. For example, Baron-Cohen and colleagues [34] developed the Sally–Anne task to compare false belief understanding in typically developing children and in children with autism. This task features a scene with two dolls, wherein one doll places a marble into a basket and leaves the scene. A second doll then moves the marble into a second location without the first doll’s knowledge. Children who demonstrate false belief understanding report that the first doll will look for her marble in its original location, whereas children who have not yet acquired this skill will report that the doll will look in its current location.

Affective perspective taking refers to identifying another’s emotions in situations where they are conflicting (different) and concordant (same) to one’s own emotions [37]. Previous research investigated young children’s ability to predict the emotions of a puppet in scenarios where the puppet’s target emotion was either the same or different from a child’s anticipated emotion [11,38,39] or via vignettes where a child considers their own feelings and the feelings of their best friend [37].

Cognitive and affective perspective taking skills have been evaluated in typically developing children through a variety of observational and experimental protocols. Though the results indicate that children most often acquire these skills by age 6 or earlier, there are no universally accepted criteria or cutoff scores used to compare an individual child’s performance within the 3–6-year age range [33,40]. Additionally, findings across studies are mixed as to whether cognitive and affective perspective taking develop simultaneously or follow related but distinct trajectories [11,24,41]. There is some evidence to suggest that these social cognitive skills develop in relation to both age and language ability, with cognitive and affective perspective taking often correlating with stronger language ability [11].

#### The relevance of perspective taking to stuttering.

Considering prior research suggesting children with typical development acquire cognitive and affective perspective taking by age 6 or earlier [33,40], young children who stutter may similarly develop the ability to take others’ perspectives within the same period as stuttering onset, initial stuttering awareness, and early reports of communication attitude. If children who stutter develop cognitive or affective perspective taking during their preschool- and kindergarten-years, they may be aware of family members’ and others’ (positive or negative) thoughts and feelings about their stuttering.

It is possible this awareness could contribute to a child’s initial communication attitude, their attitude regarding stuttered speech specifically, or their identity as a child who stutters, because children often view their skills through the eyes of their caregivers [8,9,42–44]. For example, outside of stuttering, caregivers and parents often given similar appraisals of a child’s academic ability and athleticism [8], and caregiver perceptions of a child’s skills in math and English have a stronger influence on a child’s self-perceptions compared to objective feedback such as a child’s grades [9]. Caregiver-child relationships are also significantly related to a child’s reported self-worth [43]. Relatedly, within stuttering, older children reflect on early stuttering experiences and report they were often aware of their stutter’s impact on their family, as well as their family’s worry regarding school and vocational achievements [45]. However, to date, research specific to a child’s perspective of their stuttering in early childhood has focused on communication attitude [4,13–16].

Nevertheless, one of the more commonly used tools to assess communication attitude in children who stutter requires perspective-taking. The *Communication Attitude Test for Preschool and Kindergarten Children Who Stutter (KiddyCAT)* is a 12-item self-report measure [12] used with children 3–6 years old. Individual KiddyCAT items represent components of attitude development, including evaluations of affect (e.g., Do you like to talk?), cognition (e.g., Do you think that talking is difficult?), and behavior (e.g., Do you talk well with everybody?) [21–23]. Taken together, these items are purported to provide insight into the individual’s self-perception of their “speech difficulty” [25]. Of interest to the present study, the KiddyCAT also includes two questions that require young children to reflect on how others’ feel about their communication. Specifically, the KiddyCAT asks them to consider, “do mom and dad like how you talk?” and, “do other people like how you talk?” [12]. Responding to these questions requires perspective taking, a capacity critical to social cognition and a relatively unexplored developmental domain in childhood stuttering.

Recently, Winters and Byrd [46] provided preliminary data (*n* = 44; ages 2–10 years) suggesting that cognitive perspective taking is one social cognitive skill that children who stutter acquire on a similar developmental trajectory to children with typical development, but no published studies have investigated perspective taking in children who stutter within the critical developmental period between ages 3 and 6, nor has any study investigated perspective taking as it relates to communication attitude. Such insight is needed to elucidate the factors that may contribute to caregiver-proxy – child communication attitude agreement in young children who stutter, particularly given that speech-language pathologists often formally and/or informally rely on caregiver perspectives when determining the child’s attitudes toward their communication [7]. Furthermore, with only a few of the items on the KiddyCAT requiring perspective taking and with this instrument being limited in the total number of items required, there is a need to explore item by item caregiver-proxy – child communication attitude agreement to better understand if the agreement reported is influenced by the child’s ability to think or feel what their caregiver may be thinking or feeling. Moreover, if perspective taking skills are required to provide reliable and valid responses at least on a few items, then the scores on the KiddyCAT must be interpreted with caution relative to whether or not the child demonstrates such skills.

### Communication attitude agreement

Few studies have investigated caregiver-proxy and child reports of a child’s communication attitude or the adverse impact of stuttering. Vanryckeghem [17] administered a Dutch version of the *CAT* to 55 children who stutter (ages 6–13), 55 age-matched peers who do not stutter, and their caregivers. Caregivers of children who stutter viewed their child’s communication attitude as significantly more negative than their child’s report. Similarly, for families with a family history of stuttering, 50 caregivers rated the adverse impact of stuttering on their child (age 7–12) as significantly more severe than their child did using the *OASES* [47,48].

Most recently, Winters and Byrd [19] assessed 113 caregiver-proxy and child ratings of communication attitude for children ages 3–6 who stutter. In this study, caregivers were asked to take the perspective of their child when answering items on the *KiddyCAT* [12] and rate their confidence in their responses. Caregiver ratings tended to predict their child’s communication attitude ratings, and caregivers who reported low conflict with their child were more accurate in predicting their child’s communication attitude (i.e., caregivers who reported low conflict scored more similarly to their child on the *KiddyCAT* than caregivers who reported high conflict). Other variables, including observer-rated stuttering severity (SSI-4 scores) and caregiver confidence in their prediction, did not influence the relationship between caregiver-predicted *KiddyCAT* and child-reported *KiddyCAT* scores.

Together, Vanryckeghem [17] and Winters and Byrd [19] explored similarity in communication attitude assessment scores rather than predictors of item-by-item agreement between caregivers and children who stutter. Though these studies provide helpful preliminary information about whether caregivers have a general sense of their child’s attitudes and of stuttering’s impact on their child, they do not allow for conclusions about item-by-item agreement for individual items, particularly for those items related to perspective taking. Additionally, these prior studies did not determine whether caregiver and child scores were in any way influenced by a child’s speech, language, and/or social cognition (e.g., perspective taking) abilities.

### The present study

Given that a) perspective taking develops concurrently with the typical onset of stuttering, and b) children tend to view their abilities through the perspective of their caregivers [8,9,42–44], it is possible that young children who stutter are aware of their caregiver’s thoughts and emotions related to stuttering [49–51]. This notion is reflected in the stuttering literature, in which older children often reflect on early stuttering experiences. These children report that they were often aware of their stuttering’s impact on their family, as well as their family’s worry regarding their school and vocational achievements [45]. This, coupled with the variability observed in speech and language development for children who stutter and the data to suggest typically fluent children who present with more significant articulation deficits have more significantly negative communication attitudes, prompted the present study to account for speech and language when exploring the contribution of perspective taking on caregiver-proxy – child communication attitude agreement.

In sum, the present study provides a preliminary investigation into the perspective taking of children ages 3–6 who stutter and the influence of perspective taking on caregiver-proxy – child communication attitude agreement, while accounting for additional relevant domains of speech and language. We asked the following research questions:

RQ1: What is the cognitive and affective perspective taking of 3–6-year-old children who stutter?
RQ2: Do cognitive or affective perspective taking abilities predict caregiver-proxy – child communication attitude agreement between children ages 3–6 who stutter and their caregivers, while accounting for speech and language abilities?

## Materials and methods

Data for the present study were collected in part during the COVID-19 pandemic when face-to-face human subjects research was suspended. Participants were recruited for this study between November 4, 2020 and December 7, 2022 as part of a larger study, which was approved by The University of Texas at Austin’s Institutional Review Board (IRB ID 2015-05-0044). Caregivers provided written informed consent and parent permission for their child. The first author provided the informed consent documentation to caregivers and shared information about the study and informed consent via phone prior to the first study visit. Children provided verbal assent at the beginning of their first session after hearing a description of the study from the first author.

### Participants

One hundred eleven children who stutter and one primary caregiver per child participated in the present study (N = 222 total participants). These children and their caregivers represent 111 of 113 dyads described in Winters and Byrd [19]; two dyads were removed as they did not complete all procedures relevant to the present study. Children (*n* = 111) were between ages 3 years 0 months and 6 years 11 months (mean age = 4.98 years, standard deviation = 1.13 years). Consistent with reported male-to-female ratios for childhood stuttering [5], 82 (73.9%) of the children were male, 28 (25.2%) were female, and one preferred to self-describe, as reported by the caregivers. Children represented race and ethnicity demographics aligning with those of the United States [52]; see Table 1 for full demographic characteristics and descriptions of US-specific characteristics.

**Table 1:** Demographics for children who stutter (N = 111).

	*n* (%)
**Age**	
**3-year-olds**	21 (18.9%)
**4-year-olds**	33 (29.7%)
**5-year-olds**	30 (27.1%)
**6-year-olds**	27 (24.3%)
**Age range in years**	3.0–6.92
**Mean age in years (SD)**	4.99 (1.13)
**Gender**	
**Male**	81 (73.00%)
**Female**	29 (26.13%)
**Prefer to self-describe**	1 (0.01%)
**Race**	
**White**	63 (56.76%)
**Black or African American**	20 (18.02%)
**American Indian or Alaska** **Native**	2 (1.8%)
**Asian**	7 (6.3%)
**Two or more**	3 (2.7%)
**Did not report**	16 (14.41%)
**Ethnicity**	
**Hispanic or Latino**	16 (14.41%)
**Not Hispanic or Latino**	80 (72.01%)
**Did not report**	15 (13.51%)
Socioeconomic Status*	
**SNAP eligible**	6 (5.4%)
**Medicaid**	12 (10.81%)
**Free lunch**	12 (10.81%)
**Reduced lunch**	10 (9.01%)

*Abbreviations:* SD = standard deviation, SNAP = Supplemental Nutrition Assistance Program.

*There are multiple ways to approximate socioeconomic status in the United States. In this study, we report 4 commonly used variables to capture low income or low socioeconomic status: eligibility for the Supplemental Nutrition Assistance Program (i.e., SNAP, which provides funding for low-income families to supplement their grocery bill), eligibility for Medicaid (i.e., free or low cost health coverage for low-income individuals and families), and eligibility for free or reduced lunch at school for children from low-income families. The qualifications for these metrics vary across each of the 50 states in the United States.

Though all caregivers reported that English was the primary language for their child, 33 children were exposed to additional languages: Spanish (18, or 16.2%), Mandarin (4, or 3.6%), French (3, or 2.7%), Vietnamese (2, or 1.8%), Arabic (2, or 1.8%), Persian (1, or 0.9%), Korean (1, or 0.9%), Urdu (1, or 0.9%), and Nepali (1, or 0.9%). Most of these children were reported to have low proficiency in their non-English language (i.e., understood or used only a couple of words), but two were reported as highly proficient, one in Arabic (proficiency rating 9/10) and one in Korean (proficiency rating 8/10).

The average caregiver age was 38 years old (standard deviation = 5.35, range = 23–55). Caregivers reported a range of education experience, from a high school diploma to a doctorate degree. Although most caregivers were mothers (95, or 85.6%) who were married (96, or 86.5%), some identified as fathers, and some were single parents, divorced or separated, or in a domestic partnership. Table 2 reports caregiver demographics.

**Table 2:** Caregiver demographics (N = 111).

	*n* (%)
**Relationship to child**	
**Mother**	97 (87.34%)
**Father**	14 (12.61%)
**Age**	
**Range in years**	23–55
**Mean age in years (SD)**	38 (5.35)
**Did not report**	7 (6.3%)
**Total years of education**	
**Range in years**	12–24
**Mean total years (SD)**	17.39 (2.69)
**Did not report**	5 (4.5%)
**Highest degree obtained**	
**High school diploma or GED**	9 (8.11%)
**Associates or certificate**	3 (2.7%)
**Bachelors**	35 (31.53%)
**Masters**	34 (30.63%)
**Doctorate**	20 (18.02%)
**Did not report**	10 (9.01%)
**Marital status**	
**Single parent**	10 (9.01%)
**Parents are married**	93 (83.78%)
**Parents are divorced or** **separated**	3 (2.7%)
**Parents are in domestic** **partnership**	2 (1.8%)
**Did not report**	3 (2.7%)

*Abbreviations:* SD = standard deviation, GED = General Educational Diploma.

#### Recruitment.

Families seeking an evaluation or treatment for their child who stutters were contacted through waiting lists and clinical referrals from SLPs at universities, schools, and private practices in the United States. All children who stutter and their caregivers were invited to participate in the present study as part of a virtual stuttering evaluation. Incentives for participation included $30 for families’ time and participation, a written summary of the child’s performance, and a 30–45-minute feedback session for caregivers to review their child’s performance and recommendations with two SLPs. Families were also offered an invitation to participate in an intervention study or a free professional consultation with an SLP in the child’s local community (i.e., an SLP practicing in the child’s school, a nearby private practice, or other setting) to assist in intervention planning.

#### Inclusionary and exclusionary criteria.

Families were required to live in the United States and have access to a device with internet connection. Adults were eligible to participate if they were a) a primary caregiver to a child in the present study and b) used and understood oral and written English. Children who stutter were eligible to participate in the present study if they were a) between the ages of 3 years 0 months and 6 years 11 months, b) spoke and understood English, c) did not have concerns for or a confirmed concomitant diagnosis for a neurodevelopmental disability with known potential differences in social cognition (e.g., autism), and d) had no prior experience with intervention programming at the second author’s university.

Stuttering was confirmed via at least one of the following methods: a caregiver identifying their child as a child who stutters, observation of stuttering by an SLP, and caregiver report of a prior clinical diagnosis of stuttering made by an SLP [19,53,54]. The speech and language skills of the children who stutter freely varied with population estimates, given the high prevalence of co-occurrence of speech and language disabilities (e.g., 7–46%) [31,55].

### Procedure

Data were collected within the context of a virtual stuttering evaluation across two Zoom sessions. Telepractice considerations and specific procedures for child and caregiver participants are detailed in Winters and Byrd [19]. Here, we describe the procedures and measures specific to the present study’s research questions.

#### Child protocol—stuttering, speech, language.

During session one of two, children completed the *Communication Attitude Test for Preschool and Kindergarten Children Who Stutter* (*KiddyCAT)* [12] and the perspective taking measures described below. These tasks were completed in a randomized order. The *KiddyCAT* [12] was administered via Zoom. Children were instructed to answer yes or no to two practice items and 12 scored items related to their attitudes about communication, where six scored items affirmed a positive communication attitude and six scored items affirmed a negative communication attitude. The item “Do mom and dad like how you talk?” was adjusted as needed to align with the child’s family structure (e.g., edited to “Does mom like how you talk?” for a single-parent household).

During session two, they completed the *Goldman Fristoe Test of Articulation—3*^*rd*^ *Edition (GFTA-*3) [56] *Sounds-in-Words* subtest and the *Clinical Evaluation of Language Fundamentals—Preschool 3*^*rd*^ *Edition (CELF-P3)* [57] Core Language subtests (Sentence Comprehension, Word Structure, Expressive Vocabulary). The purpose of these tests was to assess their speech sound production and expressive and receptive language, respectively.

#### Child protocol—social cognition.

Children completed cognitive and affective perspective taking tasks during the first of two Zoom sessions. The cognitive perspective taking protocol consisted of two tasks that required making inferences about others’ thoughts and beliefs. This protocol included one first-order and one second-order false belief task adapted from Baron-Cohen’s [34] original Sally–Anne task.

#### Cognitive perspective taking.

The original Sally–Anne task included several manipulatives: two dolls (Sally and Anne), a basket, a box, a door, and a marble. In the first-order task, the researcher enacts a scene where Sally places a marble into her basket and leaves the room, closing the door behind her. After Sally is out of the scene, Anne takes Sally’s marble and moves it into her own box. To conclude the scene, Sally opens the door and returns to the room. Afterward, participants answer two general knowledge questions (e.g., “Where is the marble?”) and one question targeting first-order false belief, or the ability to take the perspective of one other person (e.g., “Where will Sally look for her marble?”). Participants respond verbally or by pointing, earning a “1” for every correct response. A score of 3 would indicate a child demonstrated comprehension of the scene (2) and first-order false belief (1).

The original second-order task included a similar scene enacted by a researcher, with one change. This time, Sally looks through the keyhole and watches as Anne moves the marble from the basket to the box. At the conclusion of this scene, participants answer three questions, including general knowledge questions and questions targeting second-order false belief, or the ability to take the perspective of another person taking a second person’s perspective (e.g., “Where does Anne think Sally will look for her marble?”). Like the earlier scene, participants respond verbally or by pointing and earn a “1” for every correct response. With three questions per scene/task, participants earn up to a total of 6 possible points (3 points per task). A score of 6 would indicate a child demonstrated both comprehension of each scene (i.e., by answering general knowledge questions about the details of the scene) and first- and second-order false belief, whereas a score below 6 would indicate a combination of comprehension and/or false belief understanding.

**Fig 1:**
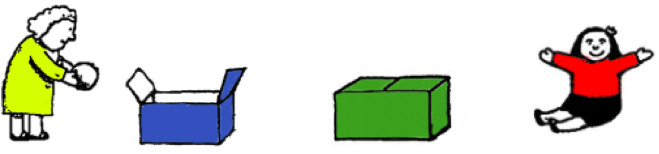
Example visual from the Sally Anne task adapted for Microsoft PowerPoint [58].

The adapted cognitive perspective taking protocol for the present study followed the procedures proposed by Sheskin and Keil [58]. Specifically, participants viewed the protocol in scenes within Microsoft PowerPoint rather than face-to-face with manipulatives, and the scene featured two different colored boxes rather than a box and basket (See Fig 1). Participants responded verbally or by pointing with their computer mouse or trackpad, with the same scoring criteria as the original version.

#### Affective perspective taking.

In the present study, the affective perspective taking protocol included two sections adapted from Harwood and Farrar [37]. In the first section, participants completed an affective labeling task to confirm understanding of the emotion vocabulary needed to complete the perspective taking section. Participants labeled emotions from a researcher’s face expressing “happy” (i.e., a smiling face) and “sad” (i.e., a frowning face).

In the second section, a researcher read aloud 12 vignettes about the participant and their best friend (see S1 Appendix). Each participant identified the name of their best friend for the researcher to use throughout the vignettes, and a caregiver or family member verified this name. Best friend names were verified to ensure the name was of a similar-aged human to fit within the context of the vignettes, as opposed to the name of an adult or pet. After each vignette, participants were asked which emotion (happy or sad) best described how *they would feel* and which emotion best described how *their friend would feel*. Vignettes featured four expected emotional outcomes: same happy (i.e., both the participant and their friend are expected to feel happy); same sad; participant happy, friend sad; and participant sad, friend happy. Each vignette was counterbalanced so that no emotion combination occurred in consecutive vignettes, and vignettes were administered in the same order across participants. Each correct response earned a “1,” for 12 possible points. Though all items elicit some form of affective perspective taking, the different emotion outcomes demonstrate a child’s ability to distinguish separate feelings for themself and their best friend and the same emotion outcomes demonstrate a child’s ability to understand shared feelings between themself and their best friend.

### Caregiver protocol

Caregivers completed a case history for their child and an adapted version of the *KiddyCAT* (abbreviated here as *C-KiddyCAT*), which was piloted and revised based on caregiver feedback (N = 20) [19]. This measure was completed asynchronously via Qualtrics before the child’s Zoom sessions. For this measure, caregivers were instructed to answer the 12 original *KiddyCAT* items from their child’s perspective. Specifically, they were asked, “I want you to answer these questions as if you were your child. In other words, I am asking you to take your child’s perspective and answer as they would, if asked the same questions.”

Scoring for the original 12 *KiddyCAT* items was the same as the original measure, meaning a researcher summed the number of responses indicative of a negative communication attitude. This resulted in a *C-KiddyCAT* score that represented the caregiver’s perception of their child’s communication attitude.

#### Analysis plan

An a priori power analysis sample size calculation for a medium effect with alpha set to.05 and power to.8 for all analyses yielded a sample size of 103 caregiver–child dyads needed for linear regression with up to 7 predictors, which are described below (total N = 111 dyads). All data were collected via Qualtrics and exported into Microsoft Excel for data cleaning (e.g., removing extraneous header information and abandoned responses). All statistical analyses were conducted in RStudio [59].

#### Calculating caregiver-proxy – child KiddyCAT agreement.

Total agreement scores were calculated in RStudio for each caregiver–child dyad, where 0 = no agreement and 1 = agreement for each item. The total agreement scores represented the summed items out of 12 in which a caregiver and their child provided the same response. Most items for which caregiver-child dyads earned a score of 1 suggest the caregiver accurately predicted how their child would respond. For example, in the case of a child who responds “no” to, “Do you like to talk?” and their caregiver who reports their child would respond “no” to the same question, agreement suggests caregivers are aware of their child’s communication attitude. For the two items for which children are asked to take others’ perspectives (i.e., “Do mom and dad like how you talk?” and “Do other people like how you talk?”), agreement suggests children may be aware of others’ attitudes toward their communication and that caregivers may be aware of their child’s awareness.

#### Statistical analysis.

To determine the cognitive and affective perspective taking skills of young children who stutter (RQ1), we used descriptive statistics to summarize performance by age. Consistent with prior studies [37], we also provide one-tailed correlations with age and cognitive perspective taking, age and affective perspective taking, and both cognitive and affective perspective taking.

To predict caregiver-proxy – child communication attitude agreement scores (RQ2), we used multiple linear regression with caregiver-proxy – child agreement score as the dependent variable and the following as independent variables: cognitive perspective taking score, affective perspective taking score, *CELF-P3* Core Language standard score, *GFTA-3 Sounds-in-Words* standard score, child age (years), and time since onset of stuttering (years). Beyond the variables of interest for RQ2 (cognitive and affective perspective taking), we included speech scores (GFTA-3) as children with speech/articulation concerns have been demonstrated to also report negative communication attitudes [10]. We included language scores (CELF-P3) as language ability is relevant both to a child’s ability to complete the KiddyCAT and perspective taking measures [11]. We included age and time since onset as these are measures frequently included in analyses related to a young child’s communication attitude [20]. Multiple regression assumptions (i.e., normality of model residuals, linear model fit, heteroscedasticity) were assessed visually in RStudio and met. Outliers were identified using Cook’s distance and visual inspection and removed. Multicollinearity was assessed via variance inflation factor (VIF) and VIF values are reported.

## Results

### RQ1: What is the cognitive and affective perspective taking of 3–6-year-old children who stutter?

Cognitive perspective taking, affective perspective taking, and age were mostly significantly correlated, consistent with previous studies [24,37]. Cognitive perspective taking and age were positively correlated, *r*(111) =.27, *p* = .05; affective perspective taking and age were positively correlated, *r*(111) =.47, *p* < .01, and cognitive and affective perspective taking were positively correlated, *r*(111) =.30, *p* < .05. Performance for these measures by age are reported in Table 3 and discussed below.

**Table 3:** Descriptive statistics (N = 111).

	Min	Max	Median	Mean	SD
**APT**					
**3-year-olds (*n* = 21)**	2	11	6	6.52	3.25
**4-year-olds (*n* = 33)**	0	12	8	8.06	3.46
**5-year-olds (*n* = 30)**	1	12	10	9.63	2.54
**6-year-olds (*n* = 27)**	5	12	12	10.96	1.65
**Total (N = 111)**	0	12	10	8.9	3.20
**CPT**					
**3-year-olds (*n* = 21)**	0	6	3	2.90	1.55
**4-year-olds (*n* = 33)**	0	6	4	3.70	1.19
**5-year-olds (*n* = 30)**	1	6	4	4.00	1.23
**6-year-olds (*n* = 27)**	2	6	4	4.04	0.98
**Total (N = 111)**	0	6	4	3.71	1.28
**CELF-P3 Core Language**					
**3-year-olds (*n* = 21)**	95	133	114	113.52	10.67
**4-year-olds (*n* = 32)**	85	133	109	108.03	14.03
**5-year-olds (*n* = 30)**	66	133	104.5	100.93	16.58
**6-year-olds (*n* = 27)**	73	128	97	99.41	13.69
**Total (N = 110)**	66	133	108	105.03	14.96
**GFTA-3 Sounds-in-Words**					
**3-year-olds (*n* = 21)**	84	117	100	99.48	10.35
**4-year-olds (*n* = 33)**	59	125	100	96.24	14.58
**5-year-olds (*n* = 30)**	60	117	89.5	93.5	13.80
**6-year-olds (*n* = 27)**	40	117	95	90.07	22.67
**Total**	40	125	97	94.61	16.24
**KiddyCAT Agreement**					
**3-year-olds (*n* = 21)**	4	11	8	7.81	1.86
**4-year-olds (*n* = 33)**	3	12	8	7.91	2.14
**5-year-olds (*n* = 30)**	3	12	8	7.77	2.40
**6-year-olds (*n* = 27)**	4	12	9	8.33	2.20
**Total**	3	12	8	7.95	2.16

*Abbreviations:* APT = affective perspective taking, CPT = cognitive perspective taking, CELF-P3 = Clinical Evaluation of Language Fundamentals – Preschool 3^rd^ Edition, GFTA-3 = Goldman Fristoe Test of Articulation – 3^rd^ Edition, KiddyCAT = *Communication Attitude Test for Preschool and Kindergarten Children Who Stutter,* SD = standard deviation.

#### Cognitive perspective taking.

Cognitive perspective taking was measured based on total task score, where children earned up to 3 points for the first-order false belief tasks and up to 3 points for the second-order false belief task (total possible points = 6). The average cognitive perspective taking task score was 3.71 out of 6 (standard deviation = 1.28). Though most children (87%) earned at least 3 points, only nine (8%) earned all 6 points. These nine children included five males and four females, and they represented the full age range of the sample (i.e., one 3-year-old, two 4-year-olds, four 5-year-olds, and two 6-year-olds).

The first-order false belief task required children to take the perspective of another person (e.g., “Where will Sally look for the ball?”). The average score for the first-order false belief task was 1.9 (standard deviation = 0.85). Twenty-six (23.42%) participants earned 3 of 3 points, 57 (51.52%) earned 2 of 3 points, 19 (17.12%) earned 1 of 3 points, and 9 (8.12%) earned 0 of 3 points. Of the participants who earned 3 of 3 points, 14 (26%) were ages 5 or 6, and 12 (22%) were ages 3 or 4.

The second-order false belief task required children to answer, “Where does Anne think Sally will look for her ball?” The children’s answers revealed their ability to understand a second person’s thoughts by taking their perspective. The average score for this task was 1.81 (standard deviation = 0.72); 17 (15.32%) participants earned 3 of 3 points, 59 (53.15%) earned 2 of 3 points, 32 (28.83%) participants earned 1 of 3 points, and 3 (2.7%) participants earned 0 of 3 points. Differences in performance were distinct between younger and older children, as 12 out of 57 children ages 5–6 (21%) earned 3 of 3 points compared to only 5 out of 54 children ages 3–4 (9%).

#### Affective perspective taking.

All children accurately labeled the happy and sad faces prior to the perspective taking vignettes. The average affective perspective taking task score was 8.9 out of 12 possible points (standard deviation = 3.2, range 0–12). Most children (55.85%) earned 10, 11, or 12 points, with 28.82% of children earning 12 points. Scores were similar across the six same emotion and six different emotion vignettes: children earned a mean score of 4.55 (standard deviation = 1.72) on the same emotion vignettes and a mean score of 4.35 (standard deviation = 1.92) on the different emotion vignettes.

Performance was consistent across participant age groups. For 3-year-olds, total perspective taking scores ranged from 2–11 (mean total score = 7; standard deviation = 3.3). Total perspective taking scores ranged from 0–12 for 4-year-olds (mean total score = 8; standard deviation = 3.4). For 5-year-olds, total perspective taking scores ranged from 1–12 (mean total score = 9.6; standard deviation = 2.5). Total perspective taking scores ranged from 5–12 for 6-year-olds (mean total score = 11; standard deviation = 1.7). Descriptively, the mean score was slightly higher among older age cohorts, though the range of scores remained consistent for children ages 3, 4, and 5.

### RQ2: Do cognitive or affective perspective taking abilities predict communication attitude agreement between children ages 3–6 who stutter and their caregivers, while accounting for variation in speech sound and language abilities?

#### Descriptive statistics.

***Caregiver-proxy–child communication attitude agreement.*** Agreement scores between caregiver *C-KiddyCAT* and child *KiddyCAT* ranged from 3 to 12 (mean = 7.96, standard deviation, 2.17). Individual KiddyCAT item agreement ranged from 0.56 to 0.85. Agreement was high on the two items asking the child to take another person’s or people’s perspectives. Specifically, agreement was highest on item 3 (“Do mom and dad like how you talk?”; mean = 0.85, standard deviation = 0.36). Agreement was third highest for item 10 (“Do other people like how you talk?”; mean = 0.73, standard deviation = 0.45).

Individual KiddyCAT item agreement for the 10 items that did not ask a child to take another person’s perspective ranged from 0.56 to 0.76. Caregivers and children agreed least often on item 1 (“Do words sometimes get stuck in your mouth?”; mean agreement = 0.56, standard deviation = 0.50). Seven of these 10 items had mean agreement scores below 0.70. In contrast, item 9 had the second highest agreement of the total 12 items (“Do you like to talk?”; mean = 0.76, standard deviation = 0.43).

***Speech and language assessment.*** The average *GFTA-3* score was 94.54 (standard deviation = 16.11, range 40–125). Eight participants (7.1%) scored in the superior range (116–125), 77 (68.14%) scored within the expected range (85–115), 16 (14.16%) scored between 1 and 1.5 standard deviations below the mean (77.5–84.0), and 12 (10.62%) scored greater than 1.5 standard deviations below the mean (40.0–77.4). Children who scored below age expectations were currently receiving speech-language intervention for articulation.

The average *CELF-P3* score was 105.17 (standard deviation = 14.86, range 66–133). Twenty-five (22.32%) participants scored in the superior range (116–133), 77 (68.75%) scored within the expected range (85–115), 10 (8.93%) scored between 1 and 1.5 standard deviations below the mean (77.5–84.0), and 10 (8.93%) scored greater than 1.5 standard deviations below the mean (66.0–77.4). Data was missing for one participant. Children who scored below age expectations were either receiving speech-language intervention for receptive and/or expressive language or received a referral for this service.

#### Inferential statistics.

Multiple linear regression was used to determine the best predictor of caregiver-proxy – child *KiddyCAT* agreement. Independent variables in the model included total affective perspective taking score, total cognitive perspective taking score, child age, time since onset of stuttering, *GFTA-3 Sounds-in-Words* standard score, and *CELF-P3* Core Language standard score. The overall model showed significant prediction of caregiver-proxy – child communication attitude agreement (*F*_(6, 96)_ = 6.32, *p* < .05) with an *R*^2^ of.28. The strongest predictors of caregiver-proxy – child communication attitude agreement were the *GFTA-3 Sounds-in-Words* standard score (*b* = 0.04, *t*_(96)_ = 4.22, *p* < .05) and age (*b* = 0.71, *t*_(96)_ = 2.8, *p* < .05). Cognitive and affec*t*ive perspective taking were not statistically significant predictors of caregiver-proxy – child communication attitude agreement. See Table 4 for full regression results.

**Table 4:** Multiple linear regression predicting caregiver-proxy – child *KiddyCAT* agreement (N = 104).

	*b*	*B*	SE	*t*-value	*p*-value	VIF value
**APT**	0.09	0.14	0.07	1.35	.18	1.45
**CPT**	−0.03	−0.02	0.15	−0.17	.86	1.22
**Child age (years)**	0.71	0.40	0.25	2.8	.006**	2.75
**CELF-P3 Core Language**	0.03	0.20	0.01	2.0	.048*	1.35
**GFTA-3**	0.05	0.39	0.01	4.22	<.001***	1.17
**Time since onset (years)**	−0.51	−0.30	0.20	−2.61	.01*	1.80

*Abbreviations:* APT = affective perspective taking, CPT = cognitive perspective taking, SE = standard error.

*p < .05; **p < .01; ***p < .001 (two-tailed).

## Discussion

The present study provides a preliminary account of a) cognitive and affective perspective taking skills of children ages 3–6 who stutter and b) predictors of caregiver-proxy – child *KiddyCAT* item agreement. In this relatively large, diverse sample of children who stutter, our data suggest there is notable variability in perspective-taking skills. Specifically, though there were participants at 3-, 4-, 5-, and 6-years of age who earned the maximum cognitive perspective taking score (6), median scores suggest performance at or above chance (3–4) for each age group. Similarly, there were 3-, 4-, 5-, and 6-year-old children who earned the maximum (12) or near maximum (11) affective perspective taking score, with median data suggesting that more than half of children across age groups demonstrated affective perspective taking at a level greater than chance. Thus, these skills range from not or minimally present to emerging or more robust across the full age range (3–6 years).

Descriptively, caregiver-proxy – child *KiddyCAT* item agreement was higher for items that required perspective taking. More accurate speech sound and language performance and older age predicted greater *KiddyCAT* item agreement for children who stutter with a range of speech sound and language ability. These findings have important clinical applications and can be used to identify areas of future research.

### RQ1: Perspective taking and clinical implications

Child performance on perspective taking measures provides support that some children as young as age 3 years who stutter begin to distinguish another’s thoughts and feelings from their own. Though there was substantial variability and most children either demonstrated minimal or emerging skills, there were 3-, 4-, 5-, and 6-year-old children who earned a maximum score on cognitive perspective taking tasks. By comparison, children across the age range demonstrated stronger performance on affective perspective taking tasks, as evidenced by descriptively higher median scores for each age cohort. Children who earned no or low points in cognitive and affective perspective taking may not yet have developed these skills or may have emerging perspective taking skills that were not captured within this study’s procedures. Taken together, results from the present study align with developmental literature suggesting that some children start to perceive others’ thoughts and feelings as early as 18 months, prior to the typical onset of stuttering [32].

In specific, consistent with previous findings using similar methods with same-age children who are typically developing, correlations between perspective taking and age somewhat varied. Descriptively, correlations between age and affective perspective taking for the present study (*r* = .47) were within the broad range between Bensalah and colleagues (*r* = .18) [24] and Harwood and Farrar (*r =* .61) [37]. Correlations between age and cognitive perspective taking for the present study (*r* = .27) were somewhat weaker than reported by Bensalah and colleagues (*r* = .58) [24] and Harwood and Farrar (*r =* .47) [37]; however, this difference may be due to our virtual task administration, as Sheskin and Keil [58] reported on somewhat weaker performance of 5- and 6-year olds on a task with similar administration to the present study. Taken together, data from the present study are similar to data with typically developing children, suggesting perhaps children who stutter begin to demonstrate emerging cognitive perspective taking at a similar age as children who do not stutter [46]. This finding can reassure SLPs and researchers that individual items in the *KiddyCAT* that require a child to take another’s perspective are appropriate and accessible to at least some children who stutter as young as 3-years-old.

Current interventions designed specifically for preschool- and kindergarten-age children who stutter involve varying levels of speaking to the child openly and directly about their stuttered speech and their experience of stuttering more generally [60–63]. Our findings support caregivers, SLPs, and other providers engaging in open and neutral discussions about stuttering with children, as children can express their attitudes about stuttering and have emerging understanding about others’ perceptions of their communication. We demonstrate evidence that at least some children show emerging perspective taking skills. Therefore, fear of discussion stuttering due to concerns that children do not understand other people may have different thoughts and feelings related to stuttering is not well-founded for all children who stutter. Our findings support continued work to provide rationale for interventions targeting open communication and advocacy skills that may foster resilience in the event of a potential adverse communication experience [60,63–67]. Given the finding that even some 3-year-old children who stutter are aware of others’ thoughts and feelings, deferring conversations about stuttering until children are older may only delay useful support such as education and advocacy skills to navigate potentially adverse communication experiences. This suggestion is consistent with recommendations from research with other clinical populations who benefit from open and neutral education and advocacy skills such as in childhood cancer [68].

### RQ2: Individual *KiddyCAT* item agreement

Clark and colleagues [25] conducted a categorical data principal components factor analysis to determine specific constructs assessed by the *KiddyCAT*, with results suggesting the measure captured one dimension: perceived speech difficulty. For the present study, caregiver-proxy – child communication attitude agreement for the 12 individual items ranged from.56 to.85. Interestingly, items inquiring about *other’s perspectives* (i.e., those that required perspective taking) had descriptively higher mean agreement scores while items inquiring about *internal experiences* had lower mean agreement scores. Specifically, two of the three items with the highest agreement assess perspective taking by asking, “Do mom and dad like how you talk?” (mean agreement = .85) and “Do people like how you talk?” (mean agreement = .73). In contrast, the six items with the lowest agreement (range.56–.62) assess internal thoughts and feelings related to stuttering (e.g., “Is talking hard for you?”). The item with the lowest mean agreement (0.56) assessed the internal experience of feeling stuck (i.e., “Do words sometimes get stuck in your mouth?).

These descriptive findings align with previous research outside of stuttering that suggests caregivers are less accurate in their estimates of their child’s internal feelings [69–71]. Though these preliminary findings are descriptive, the relatively high rate of individual item agreement, coupled with the pattern of agreement across responses, suggests that a) caregivers are generally aware of their child’s communication attitude, and/or b) children are generally aware of others’ thoughts and feelings about their communication.

It is important to note, however, that these findings do not explain *why* children and caregivers have higher agreement on items requiring perspective taking (i.e., “Do mom and dad like how you talk?”). One reason may be because children predict the perspective of their caregivers based on their own observations of caregivers’ reactions to their stuttering. Alternatively, perhaps some caregiver–child dyads provided similar answers to this question because caregivers discuss their child’s stuttering at home, either directly with the child or indirectly, such as with other caregivers or family members. This may contrast with lower agreement for items focused on internal stuttering experiences, where families may be less likely to discuss these topics at the time of their child’s initial stuttering evaluation. In either case, the clinical utility of measuring child and caregiver proxy ratings of communication attitude provides a meaningful and concrete opportunity for SLPs to discuss what young children who stutter think about their stuttering with their families. Finally, perhaps some items facilitated greater item agreement due to social pressure, in which neither children nor caregivers felt comfortable sharing that someone did not like how the child spoke.

### RQ2: Predicting communication attitude agreement and clinical implications

The predictors of caregiver-proxy – child communication attitude agreement evaluated in the present study were selected because of their relevance to perspective taking and because they represent data often collected by SLPs during their initial stuttering evaluation [7]. Although the measures of cognitive and affective perspective taking completed in the present study did not predict caregiver-proxy – child communication attitude agreement in the full sample, age and speech and language ability did.

#### The role of age and speech and language ability.

Children who stutter who were older and who had higher standard scores for speech and language assessments also had caregivers whose proxy reports resulted in greater item-by-item agreement on the *KiddyCAT,* and these predictors were statistically significant compared to cognitive and affective perspective taking scores. This finding is understandable, given that some but not all items on the *KiddyCAT* reference a child’s perspective taking ability, and all items may relate to how a child’s communication profile uniquely influences their communication attitude.

Though it is difficult to disentangle how age and speech and language ability influence this agreement on their own, perhaps children who are older provide more consistent verbal feedback about their stuttering or hear more feedback from their families. Similarly, perhaps children with strong speech and language abilities receive feedback regarding their superior skills, providing an outlet for children and caregivers to discuss communication openly.

At the same time, lower *GFTA-3* and *CELF-P3* scores were associated with lower caregiver-proxy – child communication attitude agreement. One hypothesis regarding the relationship between speech ability and caregiver-proxy – child item agreement is that children with speech sound errors present with more consistent differences in their communication compared to the variable presentation of stuttering. Perhaps caregivers of these children with co-occurring speech sound disorders are even less likely to discuss their child’s communication diagnoses or concerns with the child due to the additional perceived strain on the child’s communication. Relatedly, caregivers of children who stutter with a co-occurring language disorder may be hesitant to discuss communication openly due to concerns about the child’s comprehension. As a result, clinicians should prioritize a child’s self-reported communication attitude in their clinical evaluation. When clinicians consider a caregiver’s proxy report of their child’s attitudes, discrepancies between caregiver-proxy and child reports may serve as meaningful starting places for caregiver education or as their own intervention targets.

#### The role of perspective taking.

Young children are distinct from adolescents or adults because they often view themselves through the eyes of their caregivers [8,9,42–44]. Specific to stuttering, children reflect on early memories of their family’s feelings and worry about how stuttering might adversely impact their future academic or professional success [45]. Data from RQ1 of the present study provide preliminary support that some 3–6-year-old children are not only aware of others’ emotions, but that they make a distinction between when others’ have distinct or concordant emotions compared to their own. However, it is important to note there was considerable variability in perspective taking performance among participants in this study, particularly for the youngest participants. With this in mind, clinicians may exercise additional caution interpreting the *KiddyCAT* and items requiring perspective taking for children who may not yet demonstrate these perspective taking skills.

Although cognitive and affective perspective taking were not significant predictors in our regression model, we cannot rule out perspective taking as a relevant domain in early childhood stuttering. Perhaps the tasks implemented in the present study were not sensitive enough to distinguish the presence of these skills. Regardless, when SLPs assess speakers’ reactions to stuttering, reactions to stuttering in the environment, and potential adverse impact of stuttering [7], they should consider whether perspective taking plays a role in their individual client’s experience with stuttering and communication attitude. For example, speech-language pathologists who ask caregivers, “How do you or other people react to the child’s stuttering?” and “How does your child react to those reactions?” are inherently asking about the child’s perspective taking. Children who appear aware of others’ thoughts feelings may be more aware of listener reactions to stuttering, providing additional support for discussing stuttering in an open and neutral manner with young children and their families. Though data from the present study do not support this hypothesis directly, future research in early childhood stuttering social cognition may provide more specific clinical guidance.

#### Limitations and future directions.

This study offers meaningful findings regarding the relationship between caregiver-proxy – child communication attitude agreement and child age, language, articulation, and social cognition ability for a relatively diverse clinical sample of young children who stutter who present with speech and language skills superior to, within, and below age expectations. Although this study demonstrates cognitive and affective perspective taking among children who stutter, at an age as early as 3, future work should emphasize collecting social cognition performance in face-to-face and more naturalistic contexts, as this may better estimate how social cognition—and perspective taking more specifically—plays a role in early childhood communication attitudes. Though research methods in the present study were piloted and selected for feasible administration via Zoom due to COVID-19, it is possible that other methods may also be appropriate for this age range, such as those that require specific materials/manipulatives in a face-to-face context or those that include more items than were included in the present study. Future work should consider perspective taking assessment methods with a larger number of items, as it is difficult to disentangle whether some children in the present study demonstrated emerging perspective taking skills or if their responses were due to chance. One additional methodological limitation of the present study or consideration for future studies is the potential of undetected co-occurring diagnoses. Although data for this study were collected as part of a more in-depth evaluation, it is possible the virtual nature of the assessment limited our ability to identify potential, undetected concomitant diagnoses.

Another consideration is that in the present study, cognitive and affective perspective taking were not significant predictors of KiddyCAT item agreement. Given the few number of KiddyCAT items that require perspective taking (2) relative to the total number of items that assess speech-related attitudes (12), it is not surprising results show more salient features of speech and communication (i.e., standardized speech and language assessment scores) were better predictors of overall communication attitude. However, the present study included children who stutter with speech and language scores below, within, and above age expectations. Recruitment for future studies investigating social cognition may require larger sample sizes to elucidate the role that perspective taking plays in early childhood stuttering, as the present study’s subsample of children who stutter who exhibited speech and language scores within or above age expectations (*n* = 77) does not yield enough power to conduct a meaningful subsample analysis to test whether cognitive or affective perspective taking are better predictors of caregiver-proxy – child communication attitude agreement for children with who stutter without co-occurring diagnoses. Future studies may further investigate the role of perspective taking in communication attitudes of other clinical and non-clinical samples.

Finally, although the present study includes a diverse participant pool with respect to race, socioeconomic status, and child communication profile (i.e., presence or absence of co-occurring diagnoses), this initial analysis did not allow for in-depth considerations of how these factors may influence caregiver-proxy – child communication attitude agreement. Future work with these data (and new findings) would be beneficial for exploring how cultural, linguistic, and other family-specific factors may play a role in early childhood communication attitudes and caregiver-proxy – child communication attitude agreement.

## Conclusion

To the authors’ knowledge, this is the first study to date that investigates cognitive and affective perspective taking in children ages 3–6 who stutter. Based on a relatively large, diverse sample of children who stutter and each child’s primary caregiver, our data suggest that children show substantial variability in perspective-taking at this age. Clinicians should consider these findings when interpreting caregiver and child reports of stuttering and the related experience. More specifically, findings from this study suggest clinicians should a) prioritize collecting a child’s self-reported communication attitude, b) consider discrepancies between caregiver-proxy and child-reported metrics as a meaningful starting point for education, counseling, or for a distinct intervention target to align caregiver-proxy and child reports of stuttering experiences, and, c) use caution when interpreting *KiddyCAT* items requiring perspective taking, particularly for young children who do not yet demonstrate this skillset.

## Supporting information

S1 Appendix Affective perspective taking task adopted from Harwood and Farrar (2006). (DOCX)
