# Supplementary material for: Investigating perspective taking and caregiver-proxy–child communication attitude agreement in early childhood stuttering
Source: PLoS One. 2026 Feb 12;21(2):e0339706. doi: 10.1371/journal.pone.0339706 (PMC12900327; doi:10.1371/journal.pone.0339706)
Supplement: S1 Appendix — (DOCX) [file pone.0339706.s001.docx]

# **S1 Appendix. Affective perspective taking task adopted from Harwood and Farrar (2006).**

1. You and ______ are coloring pictures together and your teacher comes over to tell you what a good job you are both doing. How do you feel? How does ______ feel?
2. You and ______ are playing with your toys and someone walks by and steps on them. Your favorite toy gets broken, but all of ______ toys are okay. How does ______ feel? How do you feel?
3. When you are out on the playground, everyone wants to play with you and no one wants to play with ______. How does ______ feel? How do you feel?
4. Someone in your class is having a birthday party. You are not invited, but ______ is invited to go. How do you feel? How does ______ feel?
5. While playing tag, you and ______ run into each other. You both fall down and scrape your knees. How does ______ feel? How do you feel?
6. You and ______ ask the teacher if you can go play on the playground. She lets you both go play on the playground. How do you feel? How does ______ feel?
7. You both make houses out of blocks and then leave to get some more blocks. When you come back, your house is still there but someone has knocked down ______ house. How do you feel? How does ______ feel?
8. You and ______ want to go outside and play a game together, but it starts to rain and you both have to stay inside all day. How does ______ feel? How do you feel?
9. You and ______ go to the toy store together and each of you finds a different toy that you want. You do not get to buy a toy, but ______ gets to buy the toy that he/she wanted. How do you feel? How does ______ feel?
10. You and ______ are playing ‘Candyland’ together. You win the game and ______ loses the game. How does ______ feel? How do you feel?
11. The teacher yells at you and ______ for not being quiet while she reads the class a story. How do you feel? How does ______ feel?
12. You and ______ see chocolate chip cookies on the table that you would both like to eat. You both get to eat the cookies. How does ______ feel? How do you feel?
